# An update on periodontic-orthodontic interrelationships

**DOI:** 10.4103/0972-124X.65445

**Published:** 2010

**Authors:** Aous Dannan

**Affiliations:** *Department of Periodontology, Faculty of Dental Medicine, Witten/Herdecke University, Witten, Germany*

**Keywords:** Orthodontic treatment, periodontium, review

## Abstract

Talking about periodontic-orthodontic interrelationships is related primarily to the 1960s, where a generalized increase in salivary bacterial counts, especially Lactobacillus, had been shown after orthodontic band placement. The purpose of this article is to provide the dental practitioner with basic understanding of the interrelationship between periodontics and orthodontics by means of representing classical studies, and, to give an update on this topic by demonstrating the most recent opinions concerning periodontic-orthodontic interrelationships. Specific areas reviewed are the ability of orthodontic treatment to afford some degree of protection against periodontal breakdown, short-term and long-term effects of orthodontic treatment on the periodontium, and some mucogingival considerations. Topics considering orthodontic treatment in periodontally compromised patients were not included in this review. While past studies have shown that orthodontic treatment can positively affect the periodontal health, recent reviews indicate an absence of reliable evidence for the positive effects of orthodontic therapy on patients’ periodontal status. Periodontic-orthodontic interrelationships are still controversial issues. However, a standard language between the periodontist and the orthodontist must always be established to eliminate the existing communications barrier, and to improve the outcomes of the whole treatment.

## INTRODUCTION

The main objective of periodontal therapy is to restore and maintain the health and integrity of the attachment apparatus of teeth. In adult patients, the loss of teeth or periodontal support can result in pathological teeth migration involving either a single tooth or a group of teeth. This may result in the development of a median diastema or general spacing of the teeth with or without incisal proclination, rotation or tipping of bicuspids and molars with the collapse of the posterior occlusion and decreasing vertical dimension. Orthodontic treatment can often correct these problems, or at least prevent them from progressing. Additionally, orthodontic therapy can facilitate management of several restorative and aesthetic problems relating to fractured teeth, tipped abutment teeth, excess spacing, inadequate pontic space, malformed teeth, and diastema. Orthodontic treatment may improve periodontal health in these circumstances, but it also holds some potential for harm to the periodontal tissues. Thus, orthodontic treatment can be referred to as a two-edge sword, which may be sometimes very meaningful in increasing the periodontal health status, and may be sometimes a harmful procedure, which can be followed by several types of periodontal complications. However, this issue seems to be debatable.

## PERIODONTAL TISSUE AND ORTHODONTIC FORCES

Tooth movement during orthodontic therapy is the result of placing controlled forces on teeth. Removable appliances place intermittent tipping forces on teeth while fixed appliances can create continuous multidirectional forces to create torquing, intrusive, extrusive, rotational and bodily movement.[1,2] Bone surrounding a tooth subjected to a force responds in the following manner: resorption occurs where there is pressure and new bone forms where there is tension. When pressure is applied to a tooth, there is an initial period of movement for six to eight days as the periodontal ligament (PDL) is compressed. Compression of the PDL results in blood supply being cut off to an area of the PDL and this produces an avascular cell-free zone by a process termed “hyalinization”. When hyalinization occurs, the tooth stops moving. Once the hyalinized is removed, tooth movement can occur again.[2,3]

## HOW CAN ORTHODONTIC TREATMENT AFFORD SOME DEGREE OF PROTECTION AGAINST PERIODONTAL BREAKDOWN?

A strong relationship between the abnormal positions of the teeth in the dental arch and the periodontal disorders had been previously described.[4,5] Moreover, it has been shown that the number of periodontal pathogens in the anterior sites of crowded teeth is much greater than that in the sites of aligned teeth.[6] The correction of the crowded teeth can eliminate any harmful occlusal interference which may offer a great opportunity for the development of a periodontal breakdown.[7] This data definitely supports the concept that orthodontic treatment can positively affect the periodontal health, prevent the development of periodontal diseases and offer a possible action to enhance the bone formation within the bony defects.[8,9]

Generally, during orthodontic movement, the entire periodontal attachment apparatus, including the osseous structure, PDL and the soft tissue components move together with the tooth.[10] Brown looked at the influence of uprighting molars on the periodontium in four patients. Seven months following the initiation of treatment, the associated pocketing at uprighted molars had 2.5 mm greater pocket depth reduction than the one control tooth.[8] Moreover, improvement of gingival architecture and less plaque accumulation on the uprighted teeth could be noted.

In a follow-up study on 22 patients with uprighted mandibular molars after an average of 3.5 years, it was reported that pockets on the mesial surfaces were shallower on the uprighted teeth than on the control teeth.[11] Some case reports have reported that a reduction of probing depths in intrabony defects following tooth extrusion can be achieved.[12,13] In addition, there have been reported cases of localized periodontitis in which eruption of teeth reduced probing depths.[14,15] Others have also described the benefits of forced vertical eruption in the exposure of tooth structure to facilitate prosthetic treatment in healthy periodontium.[16] The use of extrusive and intrusive forces in healthy periodontium has been studied in animals[17] with favorable results in the presence of oral hygiene. The combination of orthodontic intrusion and periodontal treatment has also been shown to improve reduced periodontal conditions in animals, provided oral hygiene is maintained and tissues are healthy.[18]

Intrusion of incisors in adult patients with marginal bone loss and deep overbite has been described with root resorption varying from 1 to 3 mm. It is suggested that intrusion is best performed with low forces (5-15 g/tooth) and in the presence of gingival health.[19] Studies have also shown that moving teeth into adjacent osseous defects, orthodontic extrusion with and without fiberotomy and labial tipping of anterior teeth can be successfully accomplished without jeopardizing the periodontal support in the presence of adequate plaque control.[10,20–26]

## ADVERSE EFFECTS OF ORTHODONTIC PROCEDURES ON PERIODONTAL TISSUE

### Short-term and long-term effects of orthodontic treatment on the periodontium

Plaque is a major etiologic factor in the development of gingivitis.[27] Results from past animal studies[28–31] done in dentitions with reduced periodontium show that in the absence of plaque, orthodontic forces and tooth movements do not induce gingivitis. In the presence of plaque, however, similar forces can cause angular bone defects and with tipping and intruding movements, attachment loss can occur.[31] In healthy reduced periodontal tissue support regions, orthodontic forces kept within biological limits do not cause gingival inflammation.[31] The most important factor in the initiation, progression and recurrence of periodontal disease in reduced periodontium is the presence of microbial plaque.[28,30] Clinical studies have demonstrated that with plaque control, teeth with reduced periodontal support can undergo successful tooth movement without compromising their periodontal situation.[32,33]

The orthodontic patient’s inability to clean adequately should be expected to contribute to the development of gingival inflammation. Gingivitis and gingival enlargement appear to be the main short-term effects of orthodontic treatments on the periodontium. It has been noted that gingival enlargement occurs after placement of a fixed appliance.[34] The condition rapidly improves within 48 hours of the appliance being removed. The increase in probing depth during orthodontic treatment has been attributed, by others, to this enlargement.[35–37] As this gingival enlargement is also seen in patients with good oral hygiene, mechanical irritation caused by the band or cement must be implicated, in addition to trapped plaque.[37,38] The risk of loss of attachment can be anticipated when such iatrogenic irritations are inevitable.[35] Results of a histological study on human periodontal tissues confirm that the application and hygiene control of orthodontic bands have to be performed with great care to avoid permanent periodontal destruction.[39]

A generalized increase in salivary bacterial counts, especially Lactobacillus, has been shown after orthodontic band placement.[40] Early increase in anaerobes and Prevotella intermedia and a decrease in facultative anaerobes were also reported.[41,42] This shift in the subgingival microflora to a perio-pathogenic population is similar to the microflora at periodontally diseased sites.[43]

The orthodontic tooth intrusion used in some orthodontic treatments is considered to be a harmful procedure which may negatively affect the periodontal tissues. A non-controlled intrusive force may be resulted in root resorption, pulp disorders,[44] alveolar bone resorption, a concentrated stress within the apical part of the ligament[45] and/or an increase in the periodontal bone defects. Intrusive movements can change the relationship between the cemento-enamel conjunction and the alveolar crest which may produce an epithelial attachment along the root. With a poor oral hygiene during an orthodontic treatment, intrusion can initiate periodontal problems. It has been shown that intrusive forces usually change the position of dental plaque from supra-gingival sites to sub-gingival sites[31] which may be resulted in the formation of infra-bony defects and loss of connective tissue attachment. An increase of sub-gingival pathogens was also noted after teeth intrusion.[46] However, only a few studies did not mention the formation of periodontal pockets after tooth intrusion.[19] Other studies[47,48] have shown that after the achievement of surgical periodontal therapy of upper teeth, the intrusive forces did not show any negative effects on the periodontium, and a reduction of probing depths was clearly noted.

Some conflict exists as to the long-term effects of orthodontic treatment on the periodontium. Two retrospective studies in adults[49,50] concluded that no significant damage occurred. In a two-year post-orthodontic study, Trossello and Gianelly compared 30 adult females following multi-banded therapy with 30 age-matched control individuals.[51] They found that orthodontically treated patients had a higher prevalence of root resorption (17% vs. 2%) although there was a lower prevalence of mucogingival defects (5% vs. 12%). This root resorption was most common in the maxillary incisors followed by mandibular incisors. Radiographic crestal bone levels in 104 adult patients, who had completed orthodontic therapy at least 10 years previously, were shown in a cross sectional study to be no different to 76 matched control subjects.[52] However, Alstad and Zachrisson[53] indicated that up to 10% of 38 children had significant loss of attachment (mean 1-2 mm) in two years. In adults, it thus appears that apart from root resorption, orthodontic treatment has minimal detrimental effects on the health of the periodontium in both the short- and long-term.

In 1999, Sanders[54] conducted a review of the evidence-based literature in the fields of periodontics and orthodontics to clarify the relationship between orthodontic tooth movement and various types of common periodontal disorders; it has been shown that the literature on orthodontic tooth movement, as it relates to periodontal disease, showed that proper orthodontic treatment in patients with excellent oral hygiene and the absence of significant periodontal disorders should not pose any significant periodontal risk. In the presence of poor oral hygiene, however, and under circumstances of certain types of periodontal disorders, fixed orthodontic appliances and tooth movement can contribute to significant deleterious periodontal consequences.

## MUCOGINGIVAL CONSIDERATIONS

An adequate amount of attached gingiva is necessary for gingival health[55] and, therefore, to allow appliances (functional or orthopedic) to deliver orthodontic treatment without causing periodontal complications. With labial bodily movement, incisors showed apical displacement of the gingival margin. Loss of connective tissue occurred where inflammation was present.[56] Therefore, if the tooth movement is expected to result in a reduction of soft tissue thickness and an alveolar bone dehiscence may have occurred in the presence of inflammation, gingival recession is a risk.

Trossello and Gianelly[51] found in their retrospective study of orthodontically treated adults, a low prevalence of mucogingival defects (5%). Other clinical studies[57,58] have shown that a narrow band of gingiva is capable of withstanding the stress caused by orthodontic forces. Results from an experimental study[44] indicate that as long as the tooth is moved within the envelope of the alveolar process, the risk of harmful side-effects on the marginal soft tissue is minimal. Thin, delicate tissue is far more prone to exhibit recession during orthodontic treatment than in normal or thick tissue. If a minimal zone of attached gingiva or thin tissue exists, a free gingival graft that enhances the type of tissue around the tooth helps control inflammation. This should be done before any orthodontic movement is begun.[59–61]

Tooth extraction is considered a frequently needed procedure when planning most orthodontic treatments, especially those which aim at correcting the insufficient space disorders in the upper and the lower jaws and/or some other aesthetic and occlusal problems. The first premolars – and sometimes the second premolars – in either the upper or the lower jaw are usually the first choice when extraction becomes a part of the whole orthodontic treatment plan. Gingival invaginations are defined as superficial changes in the shape of gingiva which arise after moving the teeth orthodontically in order to close the spaces resulted from extraction procedures.[62] Gingival invaginations were noted in 35% of cases after orthodontic space closure procedures.[63] They vary from slight fissures located in the keratinized gingiva to deep gaps crossing the interdental papilla buccally or lingually through the alveolar bone deeply.[64,65] Histological and histo-chemical specimens taken from sites of gingival invagination showed hypertrophy in the epithelial and the connective tissues, and sometimes, loss of gingival collagen.[63,66] The real reason for gingival invaginations is still unknown. One expected reason could be the break-up of the continuity of the fiber models within the gingiva, and also the movement of the root.[67] However, other studies suggest the gingival peeling as a reason for such changes.[68] Since gingival invaginations may offer good sites in which dental plaque can be easily embedded, researchers considered these changes in the gingiva as risk factors for the periodontal tissue disorders during orthodontic treatment.[69]

The gingival recession has been shown to be a common adverse effect during and/or after the orthodontic treatment. This effect has been noted more frequently while using buccal orthodontic movements.[44] If teeth that have thin tissue are going to be moved lingually, there is a potential for the tissue to move coronally and become thicker.[70] If no orthodontic treatment is planned for children adolescents, areas of thin gingival tissues should be monitored only periodically as the width of the attached gingiva generally increases with normal growth from the mixed to the permanent dentition.[71]

It has been shown that most cases of gingival recession which occur during an orthodontic treatment occurred in the regions of the anterior upper and lower teeth.[50,52,72,73]

An expected relationship could be established between (Tipping) orthodontic movements and gingival recession. However, this relationship is still, to date, controversial. In a study of Batenhorst,[20] gingival recessions and bone dehiscences after orthodontic tipping of the lower incisors in monkeys had been recorded. In other studies, no real cases of gingival recession or mucogingival defects had been recorded after orthodontic tipping of the incisors.[26,70,74,75]

## RECENT ASPECTS IN PERIODONTIC-ORTHODONTIC INTERRELATIONSHIPS

Generally, the main reasons routinely cited to justify the provision of orthodontic treatment are improvement of facial and dental aesthetics and of dental health and function. However, association between malocclusions and periodontal condition is still controversial.

Ngom and co-workers[76] found significant correlations between malocclusions and periodontal condition and suggested that malocclusions are risk markers for periodontal diseases. However, a real inference about a cause/effect relationship between malocclusions and periodontal condition in this study was not possible.

A review of the literature conducted by Van Gastel[77] showed contradictory findings on the impact of malocclusion and orthodontic appliances on periodontal health, since only a few studies reported attachment loss during orthodontic treatment. It has been suggested that this contradiction may be partly due to the selection of materials and differences in the research methods employed. However, our previous studies showed that orthodontic treatment in general does not have any negative effects on the periodontal tissues when a high level of oral hygiene is maintained.[78,79]

Actually, between the year 1964 and 2007, sufficient studies had been conducted in terms of orthodontic treatment and possible related periodontal changes. Thus, it sounds plausible to extract evidence-based conclusions from those studies by means of systematic reviews.

In 2008, Bollen[80] conducted two systematic reviews to address the following questions: does a malocclusion affect periodontal health, and does orthodontic treatment affect periodontal health? The first review found a correlation between the presence of a malocclusion and periodontal disease. Subjects with greater malocclusion have more severe periodontal disease. The second review identified an absence of reliable evidence on the effects of orthodontic treatment on periodontal health. The existing low-quality evidence suggests that orthodontic therapy results in small detrimental effects to the periodontium. It has been suggested that the results of both reviews do not warrant recommendation for orthodontic treatment to prevent future periodontal problems, except for specific unusual malocclusions.

Another systematic review of controlled evidence related to the same last author[81] suggested that orthodontic therapy was associated with 0.03 millimeters of gingival recession, 0.13 mm of alveolar bone loss and 0.23 mm of increased pocket depth when compared with no treatment, and it was concluded that the effects of orthodontic therapy on gingivitis and attachment loss were inconsistent across studies. According to these recent reviews, it could be concluded that an absence of reliable evidence for the positive effects of orthodontic therapy on patients’ periodontal status seems to be indicated.[82]

On the other side, it seems that there still are studies that give the orthodontic treatment positive points regarding periodontal health. Gray and McIntyre[83] conducted a systematic literature review to determine the effectiveness of orthodontic oral health promotion (OHP) upon gingival health, and it has been found that an OHP program for patients undergoing fixed appliance orthodontic treatment produces a short-term reduction (up to 5 months) in plaque and improvement in gingival health.

A recent study of Thornberg and co-workers[84] aimed to document and investigate changes in periodontal pathogen levels before, during, and after orthodontic treatment in adolescents, eight pathogens were examined; *Actinobacillus actinomycetemcomitans* (AA), *Porphyromonas gingivalis* (PG), *Prevotella intermedia* (PI), *Tannerella forsythia* (TF), *Eikenella corrodens* (EC), *Fusobacterium nucleatum* (FN), *Treponema denticola* (TD), and *Campylobacter rectus* (CR). It has been shown that for six (PI, TF, EC, FN, TD, CR) of the eight pathogens, the percentages of subjects with high pathogen counts increased significantly after six months of fixed appliance treatment, but these returned to pretreatment levels by 12 months of orthodontic treatment. No pathogen level was significantly higher after 12 months of orthodontic treatment, and orthodontic treatment was found to be significantly protective for half of the pathogens (EC, FN, TD, and CR) post-treatment. It was concluded that orthodontic treatment with fixed appliances does not increase the risk of high levels of these periodontal pathogens.

The existing evidence, in general, does not seem to support the claim that orthodontic therapy results in overall improvement in periodontal health.

## CONCLUSION

Until 1970s, periodontists had no evidence to indicate that orthodontic treatment would either enhance or detract from the periodontal health of the patient or that it would increase or decrease the longevity of the teeth. At that time, many periodontists believed that much of the orthodontic treatment was undertaken on an esthetic or empirical basis. Nowadays, it is accepted that proper emphasis on plaque control procedures prior to initial orthodontic banding, may well minimize the inflammatory lesion often found during therapy. Although past studies have shown that orthodontic treatment can positively affect the periodontal health, recent reports and today’s existing evidence do not seem to agree with the claim that orthodontic therapy results in general improvement in periodontal health. Periodontic-orthodontic interrelationships are still controversial issues. However, in addition to the need for research in this field, it is important to emphasize that a standard language between the periodontist and the orthodontist must always be established to eliminate the existing communications barrier, and therefore, to improve the outcomes of the whole treatment.

## References

[1] Lindhe J (1989). Textbook of clinical periodontology.

[2] Proffit WR, Fields HW (1993). Contemporary orthodontics.

[3] Reitan K, Graber X, Swain BF (1985). Biomechanical principles and reactions. Current orthodontic concepts and techniques.

[4] Ashley FP, Usiskin LA, Wilson RF, Wagaiyu E (1998). The relationship between irregularity of the incisor teeth, plaque, and gingivitis: A study in a group of schoolchildren aged 11-14 years. Eur J Orthod.

[5] Bjornaas T, Rygh P, Boe OE (1994). Severe overjet and overbite reduced alveolar bone height in 19-year-old men. Am J Orthod Dentofacial Orthop.

[6] Lindhe J, Svanberg G (1974). Influence of trauma from occlusion on progression of experimental periodontitis in the beagle dog. J Clin Periodontol.

[7] Gazit E, Lieberman M (1978). The role of orthodontics as an adjunct to periodontal therapy. Refuat Hapeh Vehashinayim.

[8] Brown IS (1973). The effect of orthodontic therapy on certain types of periodontal defects. I. Clinical findings. J Periodontol.

[9] Diedrich P (2000). Periodontal relevance of anterior crowding. J Orofac Orthop.

[10] Berglundh T, Marinello CP, Lindhe J, Thilander B, Liljenberg B (1991). Periodontal tissue reactions to orthodontic extrusion: An experimental study in the dog. J Clin Periodontol.

[11] Kraal JH, Digiancinto JJ, Dail RA, Lemmerman K, Peden JW (1980). Periodontal conditions in patients after molar uprighting. J Prosthet Dent.

[12] Ingber JS (1974). Forced eruption: I: A method of treating isolated one and two wall infrabony osseous defects-rationale and case report. J Periodontol.

[13] Ingber JS (1976). Forced eruption: Part II: A method of treating nonrestorable teeth-Periodontal and restorative considerations. J Periodontol.

[14] Everett FG, Baer PN (1964). A preliminary report on the treatment of the osseous defect in periodontosis. J Periodontol.

[15] Goldstein MC, Fritz ME (1976). Treatment of periodontosis by combined orthodontic and periodontal approach: Report of case. J Am Dent Assoc.

[16] Guilford HJ, Grubb TA, Pence DL (1984). Vertical extrusion: A standardized technique. Compend Contin Educ Dent.

[17] Melsen B (1986). Tissue reaction following application of extrusive and intrusive forces to teeth in adult monkeys. Am J Orthod.

[18] Melsen B, Agerbaek N, Eriksen J, Terp S (1988). New attachment through periodontal treatment and orthodontic intrusion. Am J Orthod Dentofacial Orthop.

[19] Melsen B, Agerbaek N, Markenstam G (1989). Intrusion of incisors in adult patients with marginal bone loss. Am J Orthod Dentofacial Orthop.

[20] Batenhorst KF, Bowers GM, Williams JE (1974). Tissue changes resulting from facial tipping and extrusion of incisors in monkeys. J Periodontol.

[21] Karring T, Nyman S, Thilander B, Magnusson I (1982). Bone regeneration in orthodontically produced alveolar bone dehiscences. J Periodontal Res.

[22] Kozlovsky A, Tal H, Lieberman M (1988). Forced eruption combined with gingival fiberotomy: A technique for clinical crown lengthening. J Clin Periodontol.

[23] Polson A, Caton J, Polson AP, Nyman S, Novak J, Reed B (1984). Periodontal response after tooth movement into intrabony defects. J Periodontol.

[24] Pontoriero R, Celenza F, Ricci G, Carnevale G (1987). Rapid extrusion with fiber resection: A combined orthodontic-periodontic treatment modality. Int J Periodontics Restorative Dent.

[25] van Venrooy JR, Yukna RA (1985). Orthodontic extrusion of single-rooted teeth affected with advanced periodontal disease. Am J Orthod.

[26] Wingard CE, Bowers GM (1976). The effects of facial bone from facial tipping of incisors in monkeys. J Periodontol.

[27] Loe H, Theilade E, Jensen SB (1965). Experimental gingivitis in man. J Periodontol.

[28] Ericsson I, Thilander B (1978). Orthodontic forces and recurrence of periodontal disease: An experimental study in the dog. Am J Orthod.

[29] Ericsson I, Thilander B (1980). Orthodontic relapse in dentitions with reduced periodontal support: An experimental study in dogs. Eur J Orthod.

[30] Ericsson I, Thilander B, Lindhe J (1978). Periodontal conditions after orthodontic tooth movements in the dog. Angle Orthod.

[31] Ericsson I, Thilander B, Lindhe J, Okamoto H (1977). The effect of orthodontic tilting movements on the periodontal tissues of infected and non-infected dentitions in dogs. J Clin Periodontol.

[32] Boyd RL, Leggott PJ, Quinn RS, Eakle WS, Chambers D (1989). Periodontal implications of orthodontic treatment in adults with reduced or normal periodontal tissues versus those of adolescents. Am J Orthod Dentofacial Orthop.

[33] Eliasson LA, Hugoson A, Kurol J, Siwe H (1982). The effects of orthodontic treatment on periodontal tissues in patients with reduced periodontal support. Eur J Orthod.

[34] Baer PN, Coccaro PJ (1964). Gingival enlargement coincident with orthodontic therapy. J Periodontol.

[35] Alexander SA (1991). Effects of orthodontic attachments on the gingival health of permanent second molars. Am J Orthod Dentofacial Orthop.

[36] Kloehn JS, Pfeifer JS (1974). The effect of orthodontic treatment on the periodontium. Angle Orthod.

[37] Zachrisson BU, Zachrisson S (1972). Gingival condition associated with partial orthodontic treatment. Acta Odontol Scand.

[38] Boyd RL, Baumrind S (1992). Periodontal considerations in the use of bonds or bands on molars in adolescents and adults. Angle Orthod.

[39] Diedrich P, Rudzki-Janson I, Wehrbein H, Fritz U (2001). Effects of orthodontic bands on marginal periodontal tissues: A histologic study on two human specimens. J Orofac Orthop.

[40] Bloom RH, Brown LR (1964). A study of the effects of orthodontic appliances on the oral microbial flora. Oral Surg Oral Med Oral Pathol.

[41] Diamanti-Kipioti A, Gusberti FA, Lang NP (1987). Clinical and microbiological effects of fixed orthodontic appliances. J Clin Periodontol.

[42] Huser MC, Baehni PC, Lang R (1990). Effects of orthodontic bands on microbiologic and clinical parameters. Am J Orthod Dentofacial Orthop.

[43] Listgarten MA, Hellden L (1978). Relative distribution of bacteria at clinically healthy and periodontally diseased sites in humans. J Clin Periodontol.

[44] Wennstrom JL, Lindhe J, Sinclair F, Thilander B (1987). Some periodontal tissue reactions to orthodontic tooth movement in monkeys. J Clin Periodontol.

[45] Stenvik A, Mjor IA (1970). Pulp and dentine reactions to experimental tooth intrusion.A histologic study of the initial changes. Am J Orthod.

[46] Folio J, Rams TE, Keyes PH (1985). Orthodontic therapy in patients with juvenile periodontitis: clinical and microbiologic effects. Am J Orthod.

[47] Corrente G, Abundo R, Re S, Cardaropoli D, Cardaropoli G (2003). Orthodontic movement into infrabony defects in patients with advanced periodontal disease: A clinical and radiological study. J Periodontol.

[48] Re S, Corrente G, Abundo R, Cardaropoli D (2002). The use of orthodontic intrusive movement to reduce infrabony pockets in adult periodontal patients: A case report. Int J Periodontics Restorative Dent.

[49] Polson AM, Subtelny JD, Meitner SW, Polson AP, Sommers EW, Iker HP (1988 Jan). Long-term periodontal status after orthodontic treatment. Am J Orthod Dentofacial Orthop.

[50] Sadowsky C, BeGole EA (1981). Long-term effects of orthodontic treatment on periodontal health. Am J Orthod.

[51] Trossello VK, Gianelly AA (1979). Orthodontic treatment and periodontal status. J Periodontol.

[52] Polson AM, Reed BE (1984). Long-term effect of orthodontic treatment on crestal alveolar bone levels. J Periodontol.

[53] Alstad S, Zachrisson BU (1979). Longitudinal study of periodontal condition associated with orthodontic treatment in adolescents. Am J Orthod.

[54] Sanders NL (1999). Evidence-based care in orthodontics and periodontics: A review of the literature. J Am Dent Assoc.

[55] Lang NP, Loe H (1972). The relationship between the width of keratinized gingiva and gingival health. J Periodontol.

[56] Wennstrom J, Lindhe J, Nyman S (1981). Role of keratinized gingiva for gingival health.Clinical and histologic study of normal and regenerated gingival tissue in dogs. J Clin Periodontol.

[57] Coatoam GW, Behrents RG, Bissada NF (1981). The width of keratinized gingiva during orthodontic treatment: Its significance and impact on periodontal status. J Periodontol.

[58] Dorfman HS (1978). Mucogingival changes resulting from mandibular incisor tooth movement. Am J Orthod.

[59] Foushee DG, Moriarty JD, Simpson DM (1985). Effects of mandibular orthognathic treatment on mucogingival tissues. J Periodontol.

[60] Maynard JG (1987). The rationale for mucogingival therapy in the child and adolescent. Int J Periodontics Restorative Dent.

[61] Steiner GG, Pearson JK, Ainamo J (1981). Changes of the marginal periodontium as a result of labial tooth movement in monkeys. J Periodontol.

[62] Edwards JG (1971). The prevention of relapse in extraction cases. Am J Orthod.

[63] Kurol J, Ronnerman A, Heyden G (1982). Long-term gingival conditions after orthodontic closure of extraction sites: Histological and histochemical studies. Eur J Orthod.

[64] Rivera Circuns AL, Tulloch JF (1983). Gingival invagination in extraction sites of orthodontic patients: Their incidence, effects on periodontal health, and orthodontic treatment. Am J Orthod.

[65] Wehrbein H, Bauer W, Diedrich PR (1995). Gingival invagination area after space closure: A histologic study. Am J Orthod Dentofacial Orthop.

[66] Ronnerman A, Thilander B, Heyden G (1980). Gingival tissue reactions to orthodontic closure of extraction sites: Histologic and histochemical studies. Am J Orthod.

[67] Atherton JD (1970). The gingival response to orthodontic tooth movement. Am J Orthod.

[68] Robertson PB, Schultz LD, Levy BM (1977). Occurrence and distribution of interdental gingival clefts following orthodontic movement into bicuspid extraction sites. J Periodontol.

[69] Helm S, Petersen PE (1989). Causal relation between malocclusion and periodontal health. Acta Odontol Scand.

[70] Boyd RL (1978). Mucogingival considerations and their relationship to orthodontics. J Periodontol.

[71] Sperry TP, Speidel TM, Isaacson RJ, Worms FW (1977). The role of dental compensations in the orthodontic treatment of mandibular prognathism. Angle Orthod.

[72] Hall WB (1981). The current status of mucogingival problems and their therapy. J Periodontol.

[73] Pearson LE (1968). Gingival height of lower central incisors, orthodontically treated and untreated. Angle Orthod.

[74] Allais D, Melsen B (2003). Does labial movement of lower incisors influence the level of the gingival margin? A case-control study of adult orthodontic patients. Eur J Orthod.

[75] Djeu G, Hayes C, Zawaideh S (2002). Correlation between mandibular central incisor proclination and gingival recession during fixed appliance therapy. Angle Orthod.

[76] Ngom PI, Benoist HM, Thiam F, Diagne F, Diallo PD (2007). Influence of orthodontic anomalies on periodontal condition. Odontostomatol Trop.

[77] van Gastel J, Quirynen M, Teughels W, Carels C (2007). The relationships between malocclusion, fixed orthodontic appliances and periodontal disease: A review of the literature. Aust Orthod J.

[78] Dannan A, Darwish MA, Sawan MN (2005). Effect of orthodontic tooth movements on the periodontal tissues. Damascus Univ Journal for Health Sciences [Master Thesis].

[79] Dannan A, Darwish MA, Sawan MN (2008). How do the periodontal tissues react during the orthodontic alignment and leveling phase?. Virtual J Orthod.

[80] Bollen AM (2008). Effects of malocclusions and orthodontics on periodontal health: Evidence from a systematic review. J Dent Educ.

[81] Bollen AM, Cunha-Cruz J, Bakko DW, Huang GJ, Hujoel PP (2008). The effects of orthodontic therapy on periodontal health: A systematic review of controlled evidence. J Am Dent Assoc.

[82] Chung MH, Henwood RW (2009). Inconclusive evidence of the effects of orthodontic therapy on periodontal health. J Am Dent Assoc.

[83] Gray D, McIntyre G (2008). Does oral health promotion influence the oral hygiene and gingival health of patients undergoing fixed appliance orthodontic treatment? A systematic literature review. J Orthod.

[84] Thornberg MJ, Riolo CS, Bayirli B, Riolo ML, Van Tubergen EA, Kulbersh R (2009). Periodontal pathogen levels in adolescents before, during, and after fixed orthodontic appliance therapy. Am J Orthod Dentofacial Orthop.

